# Role of Protein Misfolding and Proteostasis Deficiency in Protein Misfolding Diseases and Aging

**DOI:** 10.1155/2013/638083

**Published:** 2013-11-17

**Authors:** Karina Cuanalo-Contreras, Abhisek Mukherjee, Claudio Soto

**Affiliations:** ^1^Mitchell Center for Alzheimer's Disease and Related Brain Disorders, Department of Neurology, University of Texas Houston Medical School, Houston, TX 77030, USA; ^2^Benemerita Universidad Autonoma de Puebla, 72160 Puebla, Mexico

## Abstract

The misfolding, aggregation, and tissue accumulation of proteins are common events in diverse chronic diseases, known as protein misfolding disorders. Many of these diseases are associated with aging, but the mechanism for this connection is unknown. Recent evidence has shown that the formation and accumulation of protein aggregates may be a process frequently occurring during normal aging, but it is unknown whether protein misfolding is a cause or a consequence of aging. To combat the formation of these misfolded aggregates cells have developed complex and complementary pathways aiming to maintain protein homeostasis. These protective pathways include the unfolded protein response, the ubiquitin proteasome system, autophagy, and the encapsulation of damaged proteins in aggresomes. In this paper we review the current knowledge on the role of protein misfolding in disease and aging as well as the implication of deficiencies in the proteostasis cellular pathways in these processes. It is likely that further understanding of the mechanisms involved in protein misfolding and the natural defense pathways may lead to novel strategies for treatment of age-dependent protein misfolding disorders and perhaps aging itself.

## 1. Introduction

Multiple and complex biological processes occur simultaneously in living cells. These processes must be strictly regulated in order to allow an optimal equilibrium and function. Proteins are key macromolecules, which perform a vast array of functions within living organisms, including replicating genetic material, catalyzing metabolic reactions, maintaining the cellular structure, participating in cellular signaling, immune responses, cell adhesion, cell cycle, responding to stimuli, and transporting molecules from one location to another. Life depends on the proper function of thousands of proteins, which in turn depends upon the acquisition of the correct, biologically functional folding of the protein. The cellular processes responsible for the synthesis, folding, and turnover of proteins are known as protein homeostasis or proteostasis [1]. The proteostasis network controls protein concentration, subcellular location, folding through molecular chaperone systems and folding enzymes, protein degradation mediated by the proteasome, lysosome, and autophagy, among others. Defects of proteostasis may commonly lead to aberrant folding, aggregation, and accumulation of proteins resulting in cellular damage and tissue dysfunction. 

## 2. Protein Misfolding in Disease

Currently there are at least 30 different human diseases reported to be associated with protein misfolding, where at least one particular protein or peptide misfolds and accumulates into a well-organized fibrillar structure often called amyloid [2]. The list includes various neurodegenerative diseases such as Alzheimer disease (AD), Parkinson disease (PD), Huntington disease (HD), Transmissible Spongiform Encephalopathies (TSE), and Amyotrophic Lateral Sclerosis (ALS) as well as diverse systemic disorders, such as familial amyloid polyneuropathy (FAP), Type II Diabetes (T2D), secondary amyloidosis, and dialysis-related amyloidosis [3]. 

Histopathological, genetic, and biochemical studies have provided compelling evidence for protein misfolding and aggregation as the critical event in the pathogenesis of PMDs. The relationship between protein misfolding and aggregation in PMDs first came from postmortem histopathological studies. Protein aggregates usually occur in the organs and regions essentially injured by each disorder [4]. Mutations in the genes that encode the protein component of fibrillar aggregates are genetically associated with inherited modes of many PMDs [4, 5]. The familial forms usually have an earlier onset and higher severity than sporadic cases and are associated with a more extensive burden of protein aggregates [5]. The development of transgenic animal models containing mutant forms of the human genes encoding the fibrillar protein is another evidence for the key contribution of protein misfolding to disease pathogenesis [6]. Several pathological and clinical characteristics of PMDs have been observed in transgenic models in which protein aggregates were produced. Finally, many *in vitro* studies have shown that misfolded oligomers and aggregates composed by different proteins acquire a cytotoxic activity, leading to cell death and tissue damage [7]. However, the mechanisms of cytotoxic and the molecular species responsible for cell damage are still unknown. Taken together these findings support the idea that the common cause of PMDs is the accumulation of misfolded protein aggregates. However, the final proof of this hypothesis would be to cure the disease in humans by arresting or reversing the formation and accumulation of misfolded protein aggregates. With a couple of exceptions, this goal has not been achieved so far [8].

## 3. Protein Misfolding in Aging

A major risk factor for most PMDs, in particular neurodegenerative diseases, is aging [9]. This finding suggests that aged cells and tissues are more prone to form and accumulate misfolded aggregates. Surprisingly, a putative role of misfolded proteins in the progressive decline of cellular and tissue functioning during natural aging has not been studied in detail. Nevertheless, recent reports have indicated that there is a widespread accumulation of insoluble proteins during aging in different species [9, 10]. Interestingly, there was a substantial overlap between the age-dependent insoluble proteins identified in worms and yeast [10–12]. Several of them have been implicated in PMDs [10]. However, it is unknown whether the age-dependent accumulation of insoluble proteins is a cause of cellular dysfunction resulting in aging or a consequence of the progressive decline of proteostasis. However, the fact that selective knockdown of these aggregation prone proteins increased lifespan of *C. elegans* suggests that accumulation of insoluble proteins may not be a mere consequence of aging [10]. Furthermore, compounds which are known to bind to protein aggregates or stimulate proteostasis lead to increase in lifespan when administered in *C. elegans*, supporting the role of protein aggregates in aging [13, 14]. Specific stimulation of cellular pathways involved in the removal of protein aggregates had similar positive effect in the longevity of *C. elegans*, *Drosophila melanogaster*, and *Mus musculus* [15–18]. 

**Figure 1:**
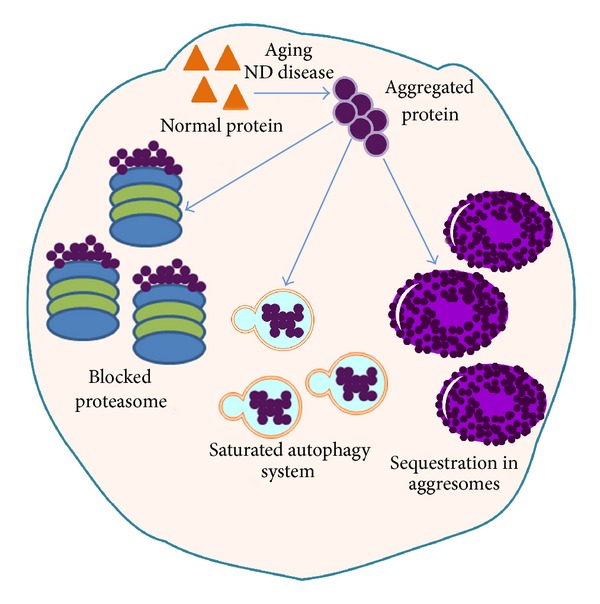
Protein aggregates formed during aging and PMDs impair diverse cellular clearance mechanisms.

Aggregation prone sequences or particular mutations may stimulate protein aggregation during aging or PMDs. However, irrespective of the cause of misfolding, stimulation of proteostasis, aiming either to prevent misfolding or to degrade aggregated proteins, may be beneficial against aging or PMDs. In this review we will summarize the role of proteostasis, including alterations in clearance mechanisms, such as proteasome, unfolded protein response, and autophagy, in disease and aging (Figure 1). As reviewed below, there is compelling evidence for the involvement of proteostasis deficiency in disease as well as natural aging. 

## 4. The Unfolded Protein Response and Its Role in PMDs and Aging

The endoplasmic reticulum (ER) is one of the major cellular organelles involved in protein homeostasis. Almost one-third of the total cellular proteins utilize the ER to attain their folded and posttranslationally modified active state [19]. Although the ER is well equipped to handle synthesis and folding of significantly high amount of proteins, genetic or environmental alterations are known to stress out the ER leading to misfolding and accumulation of proteins [20, 21]. The main mechanism by which ER combats against protein misfolding is known as the unfolded protein response (UPR) [22]. At the molecular level, UPR consists of activation of three different transmembrane proteins, including ATF6 (activated transcription factor 6), PERK (double stranded RNA activated protein kinase—like ER kinase), and IRE1*α* (inositol-requiring transmembrane kinase and endonuclease) [22]. While activated PERK blocks protein translation by phosphorylating eukaryotic translation initiation *α* (eIF2*α*), activated ATF6 (p50ATF6) acts as transcription factor to induce expression of ER-resident chaperones like BiP. When activated, IRE1 alternatively splices XBP1 mRNA. The spliced gene product induces transcription of different genes involved in the ER-associated degradation (ERAD) pathway [22]. The main goals of the UPR are to (i) shut down further protein synthesis to reduce the overload of the ER, (ii) induce ER-resident chaperones to prevent misfolding, and (iii) activate ER-associated degradation (ERAD) (IRE1*α* pathway) system to shed off misfolded protein burden using the proteasome. While temporary stress is effectively handled by the UPR, chronic stress leads to continuous accumulation of misfolded protein beyond the capacity of the UPR resulting in ER-induced suicidal response [21].

Many studies have reported the activation of the UPR in neurodegenerative diseases associated with protein misfolding which we reviewed previously [23]. Although the location of the protein aggregates in different diseases may be different, they may ultimately lead to production of chronic ER stress. In particular for PD, ALS, and TSEs, disease specific aggregates were found in the lumen of ER in the respective experimental models [24–26]. ER stress mediated cytotoxicity was also observed when cells were exposed to aggregated proteins of different sources [27–30]. Supporting this view, A*β* mediated cytotoxicity was exacerbated in cell lines compromised in specific UPR activation pathways, including PERK or XBP1 [31, 32]. Although activation of UPR in neurodegenerative disorders associated with protein aggregation is very well established, the effect of individual UPR pathways is quite complex and can be disease specific. For example, reduced expression of PERK in an ALS mouse model has been shown to accelerate the disease onset [33], leading to the idea that stimulation of PERK/eIF2*α* pathway might alleviate protein aggregate mediated ER stress. However, in a mice model of TSEs, PERK/eIF2*α* mediated sustained translational inhibition led to neuronal death which could be reversed by reinitiating the translation process [34]. Deletion of XBP1, which is the central executor of IRE1 pathway, did not influence prion disease progression in animal models [35]. However XBP1 deficiency delayed ALS and HD disease onset and progression in respective mice model by activating autophagic response [36, 37]. 

Interestingly, decline in UPR function has been shown to occur naturally during aging [38–40]. The expression level of some crucial players in the UPR, like the chaperone BiP, PDI, PERK kinase, and eIF2*α*, decreases during aging. These abnormalities shift the balance of ER stress response towards destructive pathways during aging. An optimum degree of ER stress, mild enough just to activate the protective UPR response, may be beneficial against accumulation of misfolded proteins, but a sustained and chronic activation of the UPR might have deleterious consequences [20, 41].

## 5. The Ubiquitin Proteasome System and Its Role in PMDs and Aging

The ubiquitin proteasome system (UPS) is the predominant cytoplasmic cellular network responsible for the degradation of short-lived, damaged, and abnormal proteins [42, 43]. Thus it plays a crucial role in the maintenance of cellular dynamics. Altered proteins tagged with ubiquitin are recognized by the proteasome for proteolytic degradation. A detailed mechanism of the ubiquitinylation process has been described elsewhere [42]. Proteasome is a multisubunit, barrel-shaped complex composed of 20S catalytic core particle and two 19S regulatory particles located at the edges of the core forming the 26S proteasome [44]. 

Compelling evidence has shown impaired proteasome function in neurodegenerative disorders associated with protein misfolding [45, 46]. Supporting this view, when proteasome function was decreased in adult rats using synthetic inhibitors, the animals presented Parkinson-like symptoms and degeneration of the substantia nigra pars compacta [47]. However, those results were not reproducible by a different group [48]. In autosomal recessive PD, genetic mutations in the gene encoding an ubiquitin ligase, involved in proteasomal degradation (Parkin), lead to its loss of function resulting in accumulation of damaged proteins and consequent neuronal injury [49, 50]. In a cellular model expressing truncated tau protein, reduction of proteasomal activity resulted in increase in protein aggregation. Conversely, using chemical activation of proteasome by geldanamycin, it was possible to accelerate the degradation of this intracellular misfolded protein [51]. Since optimum proteasome function is crucial for cellular homeostasis, a compromised proteasome immediately became a target in PMDs. Furthermore it was suggested that a proteasome with diminished function could eventually promote more aggregate formation leading to cellular toxicity [52]. One limitation for the proteasome to clear protein aggregates is that the large size and proteolytic stability of misfolded aggregates pose difficulties for them to enter into the proteasome chamber that has a pore size of 13 angstroms. In a recent study aggregated *β*-sheet-rich PrP was shown to decrease proteasome activity by blocking the opening of 20S proteasome [53]. Similar observation was made in case of AD where ubiquitinated and aggregated tau has been found to bind the substrate recognition site of the proteasome, leading to a steric hindrance in the entry site of the catalytic core [54]. This problem often results in jamming the proteasome, which may have deleterious consequences for proteostasis [43]. In fact, aggregates formed by different proteins have been shown to directly inhibit proteasome activity [55–58].

Generation of transgenic mice with impaired proteasome activity is extremely difficult due to the crucial function of proteasome during development. However, a conditional inactivation of an ATPase subunit (Rpt2) of the proteasome in substantia nigra's neurons showed accumulation of *α*-synuclein positive, Lewy body-like deposits followed by severe neurodegeneration [59]. Supporting further the key role of proteasome in PMDs, it has been demonstrated that a motor neuron specific deletion of ATPase subunit (Rpt3) leads to accumulation of TDP43 and FUS proteins, followed by progressive loss of motor neuron resembling ALS pathology [60]. In an attempt to study the effect of chronic proteasome inhibition, a mice model overexpressing proteasome antagonist UBB^+1^ (mutant form of ubiquitin B) was generated. Strikingly, just 20% of proteasomal inhibition was enough to produce an AD-like behavioral deficit in this mouse model [61].

Decrease in proteasomal activity with aging has also been widely reported [62–65]. An age-related decrease in proteasome activity weakens cellular capacity to remove damaged proteins and favors the development of diseases [65]. It has been recently found that a transgenic mouse model exhibiting decreased chymotrypsin-like proteasome activity had a shortened lifespan [66]. These transgenic mice accumulate damaged and oxidized proteins and presented premature aging signs as well as aggravated age-related metabolic disorders [66]. Another example that links proteasomal activity with aging came from studies performed on the longest-lived rodent, *Heterocephalus glaber*, better known as naked mole rat. The lifespan of this organism is about 30 years and they remain healthy during the major part of their life. Analysis of the three different catalytic activities of the proteasome, in comparison to mice, revealed that there is a three- and sixfold increase in trypsin-like and chymotrypsin-like proteasome activity, respectively, which promotes a highly efficient protein turnover and clearance of misfolded and damaged proteins [67]. *Heterocephalus glaber*, as well as other species including *Homo sapiens*, accumulates with age an intracellular fluorescent yellowish pigment called lipofuscin, which is a general marker of aging and is resistant to removal by degradation [68, 69]. It is mainly composed by oxidized and crosslinked proteins and in a minor extent by lipids and sugars. Similar to the protein aggregates produced during neurodegenerative disorders, lipofuscin has also been shown to inhibit proteasome and reduce the rate of protein degradation [70]. 

Preserving a balanced proteasome activity during chronological aging might be an interesting strategy to elongate lifespan and prevent age-related degenerative disorders associated with protein misfolding. Many studies have been done using model systems to evaluate the effect in aging and disease of genetic manipulation of diverse components of the proteasome (Table 1). Ectopic expression of the non-ATPase subunit (Rpn11) of the 19S regulatory particle has been shown to maintain the integrity of the proteasome and to suppress polyglutamine induced toxicity in *Drosophila melanogaster* [15]. Furthermore, Rpn11 overexpression in the adulthood was enough to significantly extend the mean lifespan [15]. Similar extension of lifespan was observed when Rpn6 subunit expression was elevated in a mutant form of the nematode *Caenorhabditis elegans* [16]. When overexpressed in wild type worms, Rpn6 had a positive effect in lifespan under mild stress. Conversely, silencing the same subunit resulted in a decreased longevity and less resistance to stress conditions [16]. The same group also reported that the homolog of RPN-6 subunit in *Homo sapiens*, PSMD-11, is naturally overexpressed in human embryonic stem cells (hESC) that do not exhibit replicative senescence, leading to a high level of proteasomal activity [71]. It seems that the PSMD11/RPN6 subunit stabilizes the interactions between the 20S and 19S proteasome resulting in a higher efficiency of proteasome assembly [72]. Taken together this evidence strongly suggests that there is a fundamental role of proteasome activity in aging and degenerative diseases associated with protein misfolding. 

## 6. Autophagy and Its Role in PMDs and Aging

Besides of the UPS, autophagy is another clearance mechanism to degrade damaged organelles and proteins [73, 74]. It involves the lysosomal degradation system and is implicated in multiple conserved pathways that regulate metabolism and longevity [75, 76]. Normally, it is activated under stress conditions, e.g., starvation, as a protective mechanism to ensure survival and cellular homeostasis by protein turnover [77]. Autophagy is classified in three different types according to the mechanism used for the capture and degradation of substrates: chaperone mediated autophagy (CMA), microautophagy, and macroautophagy [74]. In CMA, proteins that contain the pentapeptide KFERQ are recognized by the chaperone heat shock cognate protein 70 and transported to the lysosome for its hydrolysis [78]. Microautophagy refers to a process in which some portions of the cytosol are trapped directly by the lysosome without the intervention of chaperones [79]. Macroautophagy involves sequestration of damaged organelles or large protein aggregates into cargo vesicles known as autophagosomes that transport the contents to the lysosome for its degradation [80]. Although autophagy is considered to be an adaptive process, current studies suggest that a basal level of autophagy is always active and is involved in protein quality control [81–83].

A crucial role for autophagy in neurodegenerative disorders associated with protein misfolding has been recently recognized [84]. Accumulation of autophagic vacuoles has been found in different inherited forms of neurodegenerative diseases [85, 86]. Aggregation prone proteins related to AD (tau), PD (*α*-synuclein), and HD (polyQ-expanded huntingtin) are known substrates for autophagy. Furthermore, enhanced autophagy has been shown to reduce polyQ-expanded huntingtin aggregates and toxicity in different models including cells, *Drosophila*, and mice [87–90]. Inhibition of autophagy has also been reported to exacerbate protein aggregation and toxicity in these models. Similar results were obtained in *Drosophila* overexpressing AD specific mutant of tau, strengthening the involvement of autophagy in the clearance of disease specific protein aggregates [87]. In a mouse model of AD (expressing human A*β*), heterozygous deletion of beclin 1 (a protein that participates in the regulation of autophagy) resulted in a reduction of autophagy which, in turn, generated an exacerbated AD pathology, including extra- and intracellular A*β* deposition and neurodegeneration [91]. Partial recovery of autophagy by lentiviral administration of beclin 1 reduced the AD pathology. Similarly, improved clearance of A*β* aggregates was observed in mouse model of AD when autophagy was stimulated by administration of the antihistamine drug Latrepirdine [92]. Even in the absence of any disease specific proteins, central nervous system specific reduction of autophagy by conditional deficiency of *Atg7* (autophagy target gene 7) resulted in loss of pyramidal neurons in hippocampus, cortex, and Purkinje cells in the cerebellum [93]. Similar results were obtained by other groups when they genetically reduced autophagy by selective deficiency of *Atg5* or *Atg17*/FIP200 in the neurons [94, 95]. The mechanism that distinguishes nutrient dependent adaptive autophagy from basal autophagy, involved in protein quality control, is still a mystery. HDAC6 (ubiquitin-binding deacetylase, histone deacetylase-6) was identified as the central component of basal autophagy which is not involved in the autophagy activation [96]. This protein appears to play an important role in the fusion of autophagosome to lysosome where the aggregated proteins are degraded. Interestingly, HDAC6 inactivation resulted in accumulation of protein aggregates and neurodegeneration [96]. Transport of autophagosomes to lysosome is governed by the dynein motor. Motor neuron disease specific mutation in the dynein has been shown to reduce removal of aggregated proteins. Furthermore, this mutation in dynein machinery enhanced mutant huntingtin aggregation and toxicity in fly and mouse models of HD [97].

Several studies reported reduced expression of different autophagy related genes, including Atgs, in aging [98], resulting in reduced autophagy and in turn accumulation of lipofuscin. Loss-of-function mutations in Atg1, 7, and 18 and beclin 1 have been shown to reduce lifespan in *C. elegans* [98]. The fact that even in case of normal human brain aging Atg5 and 7 and beclin 1 are downregulated suggests altogether a crucial role of autophagy in aging [98]. The first hint suggesting that an enhanced autophagy may increase lifespan came from the finding that caloric restriction, which elongates lifespan in almost all species tested, induces autophagy [99]. Even more importantly, prevention of autophagy abolishes the effect of caloric restriction in different experimental models. Analysis of the genes that are upregulated during caloric restriction showed that the LIPL-4 lipase in worms may have a role in the observed longevity extension by a mechanism that possibly activates autophagic response through the production of *ω*-6 polyunsaturated fatty acids [100]. Interestingly, *ω*-6 polyunsaturated fatty acids have been found to induce autophagy and increase lifespan even in conditions that do not resemble caloric response. Moreover, in human epithelial cells autophagy was activated when the medium was supplemented with *ω*-6 polyunsaturated fatty acids [100]. These results suggest that autophagy may be a crucial target for lifespan extension. 

One of the evolutionary conserved pathways in eukaryotes that regulate autophagy is the target of rapamycin (TOR) serine-threonine kinase [101]. TOR can associate with distinct proteins and form two different complexes, TORC1 and TORC2. When TOR is active, it triggers anabolic processes that include increase in protein synthesis along with a reduction of autophagy [101]. Rapamycin, a compound discovered and isolated from a soil sample of the Chilean Easter Island, inhibits TORC1 signaling. It binds to the protein FKBP12, forming a complex that subsequently binds and inhibits TOR, leading to activation of autophagy [102]. Cumulative evidence coming from studies using rapamycin suggests a possible role of autophagy in longevity, aging, and neurodegeneration. A study performed in *C. elegans* demonstrated that rapamycin treatment as well as genetic knockdown of TORC1 signaling increased stress resistance and autophagy and had positive impact in health and lifespan [103]. The effect of rapamycin and caffeine on TOR inhibition and lifespan was also tested using *Schizosaccharomyces pombe* as a model organism. An increased longevity and decreased aging rate was observed with both compounds. However, caffeine seems to inhibit TOR at a transcriptional level rather than by a direct interaction with TOR [104]. Moreover, when encapsulated rapamycin was administered in the diet of 600-day-old mice, an extension in the median and maximal lifespan was observed [18, 105]. However, one has to be cautious while interpreting the effect of rapamycin on aging. Rapamycin also suppresses inflammation, which has positive effect on lifespan. A recent study gives insight into the role of TORC1 in the regulation of autophagy and aggregated proteins in *Saccharomyces cerevisiae* [106]. Proteins that become insoluble with age are sequestered into autophagic bodies, visible by light microscopy in aged cells. By stimulation of the autophagic machinery, using nitrogen starvation, an increase was observed in the amount of these cargo vesicles [12]. Insoluble protein accumulation was observed when TORC1 was inhibited genetically and pharmacologically, suggesting that the protein transition to insolubility and sequestration in autophagic vesicles is an intermediate process before autophagic degradation and that is regulated by TORC1 [12]. However, it was also shown that insoluble protein accumulation is not necessarily dependent on autophagic activation, indicating that TORC1 regulates both processes using different mechanisms and that most likely they act together to eliminate damaged proteins [12]. 

## 7. Aggresomes and Their Role in PMDs and Aging

Aggresomes are cytoplasmic inclusion bodies that sequester aggregated proteins [106]. Formation of aggresomes appears to be a protective response when there is an excessive accumulation of misfolded proteins that cannot be cleared by canonical mechanisms like UPS or autophagy. Aggresomes may be formed as a transient mechanism to respond to impaired proteostasis under these conditions [107]. Damaged proteins are transported through the cytoskeleton to the centrosome or the microtubule organizing center with the help of accessory proteins, where aggregated proteins reach a high local concentration leading to the formation of aggresomes. It is believed that aggresomes act as a cytoprotective method preventing the interaction of aberrant proteins with normal cellular molecules. Evidence also indicates that there is recruitment of UPS and lysosomes, suggesting that aggresomes can be digested by these two proteostasis mechanisms [108–110].

To study the role of the aggresome formation in PMDs, a yeast model expressing polyQ polypeptides was developed. In this model when aggresome formation was prevented, there was an increased proteotoxicity supporting the view of a protective mechanism [111]. It also seems that there are recognition signals, like proline-rich domains, that may target some proteins to the aggresomes. Another signal that is believed to be involved in the formation of aggresomes is the ankyrin-like repeat in synphilin 1, a protein related to PD [112]. When proteasome inhibitors were administered to cells expressing synphilin 1, the formation of aggresomes was promoted, suggesting that proteasomal inhibition may be a signal that triggers aggresome formation [112]. 

When aggresomes are formed in mitotic cells, the distribution of the aggregates becomes asymmetrical upon cell division, causing one cell to “inherit” more damaged proteins than the other [113]. Apparently the aggresomes are segregated to the daughter cells through a mechanism involving the microtubule organizing center. It has been suggested that asymmetrical distribution of cellular components, causing one cell to receive more damage than the other, leads to differential aging [114]. A similar phenomenon was observed in *D. melanogaster* neuroblasts expressing the N-terminal fragment of human huntingtin that under experimental conditions formed aggresomes [115]. Neuroblasts divided to give rise to another neuroblast and a ganglion mother cell. Neuroblasts are short-lived cells that die during the embryogenesis process whereas ganglion mother cells divide in two cells that survive during all the fly lifespan. Analysis of the segregation of the aggresomes showed that the inclusion body was always inherited to the short-lived cell (neuroblast) and the ganglion mother cell did not receive the damaged proteins. These results suggest that the formation and segregation of aggresomes could have implications for the processes of cellular differentiation and aging [115].

## 8. Concluding Remarks

It is estimated that by 2050 there will be 2 billion people aged over 60 years old. Increased vulnerability of cells to physiological and environmental stress due to loss of protein homeostasis raises disease susceptibility with aging. Therefore the process of aging itself will greatly increase the onset of different diseases. All neurodegenerative diseases and most PMDs are strictly associated with aging, suggesting a link between protein misfolding and aging. Recent studies indicate that protein misfolding and aggregation of a widespread range of proteins naturally occur with time in different species. In case of diseases associated with protein misfolding, genetic mutations drastically increase the aggregation propensity of specific proteins, leading to accelerated accumulation of protein aggregates. Initially, the clearance machinery takes care of it. However, with time, the clearance capacity is compromised either due to aging or by a direct inhibitory activity of protein aggregates, resulting in the disruption of cellular homeostasis. This generates a death cycle in which protein misfolding promoted by aging defects leads to further damage of the clearance machinery, which in turn produces more accumulation of misfolded aggregates, getting to the point that these structures cause cellular toxicity, tissue dysfunction, and disease. Boosting up the clearance machinery by genetic and pharmacological tools showed beneficial effect on lifespan and protection against neurodegenerative disorders associated with protein misfolding in different animal models. It is important to keep in mind that an optimum activity of the clearance machinery is crucial to maintain steady-state level of different proteins in the cell. Therefore, an imprudent stimulation of clearance may be harmful as well. Thus, further studies are required to understand the specific mechanism of protein misfolding, the involvement of the clearance machinery, and the development of therapeutic strategies to combat the accumulation of misfolded protein aggregates and their beneficial effect in disease and aging.

## Figures and Tables

**Table 1:** Genetic modulation of proteasome in different models and its effect on aging and disease.

Subunit deficiency/overexpression	Function	Phenotype	Model	Reference
Rpt2 inactivation	ATPase	Ubiquitin and *α*-synuclein positive Lewy like intraneural inclusion in neurons and neurodegeneration	*Mus musculus *	[59]

Rpt3 inactivation	ATPase	TDP43, FUS accumulation, basophilic inclusion bodies in neurons, locomotor impairment, loss of neurons	*Mus musculus *	[60]

*β*5t deletion	Chymotrypsin-like proteolytic activity	Shortening of lifespan, accumulation of polyubiquitinated and oxidized proteins, aggravated age-related metabolic disorder	*Mus musculus *	[66]

Rpn11 overexpression	Deubiquitination of the proteasome substrate	Extension of lifespan, suppression of polyQ induced toxicity	*Drosophila melanogaster *	[15]

Rpn6 overexpression	Stabilizing the interaction between CP and RP	Extension of lifespan under mild stress condition	*Caenorhabditis elegans *	[16]
